# Distinction of Physiologic and Epileptic Ripples: An Electrical Stimulation Study

**DOI:** 10.3390/brainsci11050538

**Published:** 2021-04-24

**Authors:** Jan Schönberger, Anja Knopf, Kerstin Alexandra Klotz, Matthias Dümpelmann, Andreas Schulze-Bonhage, Julia Jacobs

**Affiliations:** 1 Epilepsy Center, Medical Center, University of Freiburg, 79106 Freiburg, Germany; anja.knopf@uniklinik-freiburg.de (A.K.); kerstin.alexandra.klotz@uniklinik-freiburg.de (K.A.K.); matthias.duempelmann@uniklinik-freiburg.de (M.D.); andreas.schulze-bonhage@uniklinik-freiburg.de (A.S.-B.); 2 Department of Neuropediatrics and Muscle Disorders, Medical Center, University of Freiburg, 79106 Freiburg, Germany; Julia.jacobs@uniklinik-freiburg.de; 3 Faculty of Medicine, University of Freiburg, 79085 Freiburg, Germany; 4 Berta-Ottenstein-Programme, Faculty of Medicine, University of Freiburg, 79085 Freiburg, Germany; 5 Department of Paediatrics and Department of Neuroscience, Cumming School of Medicine, University of Calgary, Calgary, AB T3B 6A8, Canada; 6 Hotchkiss Brain Institute and Alberta Children’s Hospital Research Institute, University of Calgary, Calgary, AB T2N 4N1, Canada

**Keywords:** epilepsy, electroencephalography, intracranial, high-frequency oscillations, physiologic, stimulation, neocortical

## Abstract

Ripple oscillations (80–250 Hz) are a promising biomarker of epileptic activity, but are also involved in memory consolidation, which impairs their value as a diagnostic tool. Distinguishing physiologic from epileptic ripples has been particularly challenging because usually, invasive recordings are only performed in patients with refractory epilepsy. Here, we identified ‘healthy’ brain areas based on electrical stimulation and hypothesized that these regions specifically generate ‘pure’ ripples not coupled to spikes. Intracranial electroencephalography (EEG) recorded with subdural grid electrodes was retrospectively analyzed in 19 patients with drug-resistant focal epilepsy. Interictal spikes and ripples were automatically detected in slow-wave sleep using the publicly available Delphos software. We found that rates of spikes, ripples and ripples coupled to spikes (‘spike–ripples’) were higher inside the seizure-onset zone (*p* < 0.001). A comparison of receiver operating characteristic curves revealed that spike–ripples slightly delineated the seizure-onset zone channels, but did this significantly better than spikes (*p* < 0.001). Ripples were more frequent in the eloquent neocortex than in the remaining non-seizure onset zone areas (*p* < 0.001). This was due to the higher rates of ‘pure’ ripples (*p* < 0.001; median rates 3.3/min vs. 1.4/min), whereas spike–ripple rates were not significantly different (*p* = 0.87). ‘Pure’ ripples identified ‘healthy’ channels significantly better than chance (*p* < 0.001). Our findings suggest that, in contrast to epileptic spike–ripples, ‘pure’ ripples are mainly physiological. They may be considered, in addition to electrical stimulation, to delineate eloquent cortex in pre-surgical patients. Since we applied open source software for detection, our approach may be generally suited to tackle a variety of research questions in epilepsy and cognitive science.

## 1. Introduction

High-frequency oscillations (HFOs), traditionally divided into ripples (80–250 Hz) and fast ripples (250–500 Hz), are a promising marker of epileptic activity. They have not only been directly linked to seizures [1,2,3,4]; moreover, there is a by now an extensive body of evidence on the value of interictal HFOs: resecting HFO-generating tissue has been associated with seizure-free outcome [5,6,7,8], HFO rates correlated with response to electrical stimulation [9], HFOs were suppressed by antiepileptic medication [10,11], and ripples may identify patients at risk of developing epilepsy [12]. Nonetheless, HFOs are rarely analyzed in clinical routine settings. One reason may be that the visual identification is time-consuming, an obstacle that may be overcome by the increasing efficiency of automatic detectors [13,14,15,16]. Another key aspect is that ripples are likely involved in memory consolidation [17,18,19], which may open new avenues for cognitive science, but impairs specificity if ripples are analyzed in epilepsy. To develop strategies that reliably distinguish pathologic from physiologic HFOs is thus of imminent importance for researchers from both fields [20].

To design studies on potential approaches is, however, challenging, considering that most intracranial recordings are from patients with epilepsy and that in these subjects, there is both epileptic and physiologic activity. A fruitful approach has been to hypothesize that sleep influences epileptic and physiologic ripples differently. Following this concept, it was revealed that HFOs from epileptic and ‘normal’ channels are synchronized to distinct phases of sleep slow waves [21,22,23], and that they are associated with different sleep stages [24]. Moreover, systematic differences in HFO amplitude and frequency have been reported, though not always consistent and often with significant overlap [25,26,27]. A third approach has been to define epileptic ripples based on coupling to epileptic spikes. Several recent studies suggest that such ‘spike–ripples’ may indeed be superior to ripples or spikes, regarding the delineation of seizure-generating areas [28,29,30] and the prediction of seizure risk [31]. On the other hand, however, one study also found that spike-HFOs performed no better than spikes [32]. The additional value of HFOs is thus still subject to debate [33,34]. Finally, it has remained unclear whether the remaining ‘pure’ ripples, i.e., those occurring independently from spikes, are truly physiological.

We thus set out, in a retrospective analysis of subdural grid electrode recordings from patients with drug-resistant focal epilepsy, to explore whether such ‘pure’ ripples are specifically generated in ‘healthy’ brain areas, identified based on our patients’ response to electrical stimulation. Moreover, we aimed to provide additional evidence for the concept that spike–ripples are truly epileptic, which would underline that analyzing coupling to interictal spikes is indeed a promising strategy to distinguish physiologic from epileptic ripples.

## 2. Methods

### 2.1. Patient Selection

We considered all patients with drug-resistant focal epilepsy who, as part of their evaluation for epilepsy surgery, had undergone the implantation of subdural grid electrodes at the Freiburg Epilepsy Center in 2008 or 2009. From this cohort, subjects with contacts recording from the eloquent cortex, identified based on cortical stimulation, were selected. Recruitment was restricted to the specified period because afterwards a new video-EEG recording system was in use, and we aimed not to examine a mixed dataset obtained with different hardware setups. This study was approved by the Ethics Commission at the University Medical Center Freiburg (No. 69/18) and written informed consent was obtained from all patients.

### 2.2. Electrical Stimulation and Assignment of Grid Contacts

Electrode grids produced by Ad-Tech (Ad-Tech Medical Instrument Corporation, Racine, WI, USA), which were implanted after open craniotomy. Grids had 32, 48 or 64 contacts, each with a center-to-center distance of 10 mm and an exposed surface diameter of 2.3 mm. The decisions on the size of the grid, its position and the stimulation protocols were made solely by the attending physician and thus not influenced by this study. 

Cortical stimulation was performed during continuous video-EEG monitoring on all contacts, except for those that were obviously damaged, i.e., with frequent artifacts or high impedance. Pairs of electrodes were stimulated at 50 Hz with a biphasic rectangular pulse (width 0.25 ms) for 10 s. Stimulation was repeated every 30 to 60 s, with gradually increased electrical currents (max. 15 mA) until the patient had a seizure or symptoms indicating that the stimulated tissue was involved in a particular function. Typical motor symptoms like clonus, tonic movements or weakness, and various sensory phenomena like paresthesia, hallucinations, nausea or pain were documented in a stimulation protocol, which was retrospectively analyzed by our study team. To examine verbal impairment, a series of tasks was performed, which typically included an assessment of reading, serial and repetitive language, body commands and a token test. We relied on the assessment of the attending physician, i.e., on data generated independently from this study, to determine whether or not an electrode contact was located on the functional cortex.

The seizure onset zone (SOZ) was defined by contacts with a clearly ictal EEG pattern within two seconds of seizure onset. Seizure onset was defined based on EEG and semiology, as part of the clinical routine evaluation and under the supervision of the attending physician. We identified SOZ contacts based on this assessment, i.e., based on data generated independently from this study. All remaining channels were classified as ‘non-SOZ’ channels.

### 2.3. Detection of Interictal Epileptic Spikes and Ripples

Intracranial EEG was recorded with a Neurofile NT system (IT-Med, Usingen, Germany). The sampling rate was 1024 kHz and a low-pass filter with 450 Hz cut-off frequency was applied. Forehead electrodes were used as an amplifier ground. For each patient, we selected a 1 h segment of non-rapid eye movement sleep, at least two hours before and after a seizure. We took great care to ensure that this segment contained no clear or only very sparse muscle artifacts.

Interictal epileptic spikes and ripples (80–250 Hz) were identified in bipolar montages using the Delphos detector (Version 1.0.1; [16,35]) within the open source software Anywave [36]. A detailed description of the algorithm was provided in the original publications. This detector was benchmarked against previously published methods. The default settings were kept (number of voices 12, vanishing moment 20, threshold 40, oscillation width threshold 1.4, oscillation frequency spread threshold 10, spike width threshold 1.3, spike frequency spread threshold 11). Further analyses were performed with Matlab R2019b (Version 9.7; Mathworks, Natick, MA, USA). Spike–ripples were defined as spikes and ripples that co-occurred within 100 ms [32].

### 2.4. Quantifying Performance of Different Biomarkers

To quantify how reliably a biomarker could distinguish SOZ from non-SOZ channels, receiver operating characteristic (ROC) curves and their area under the curve (AUC) were computed using the Matlab built-in function ‘perfcurve’. We also determined the partial AUC (pAUC) between 100% and 85% specificity, as has been suggested in a previous study comparing the diagnostic values of spikes and high-frequency oscillations in patients with epilepsy [32]. This parameter is particularly useful if ROC curves tend to reach a plateau, reflecting that a classifier performs poorly in a distinct range of thresholds. Reproducing our colleagues’ approach, the ‘raw’ pAUC values were divided by the maximum possible area, i.e., 0.15, to obtain an index ranging from 0 to 1. It may be noteworthy that the expected AUC for random classification is 0.5, whereas the chance-level pAUC, if normalized as in our study, should only be 0.075.

### 2.5. Statistical Analysis

We did not assume that the data were normally distributed. Therefore, the median was specified as a measure of central tendency and the range as a measure of dispersion. Hypothesis tests were performed as two-tailed tests. A significance level of 5% was chosen. Paired data were compared with a Wilcoxon signed-rank test, unpaired data with a Wilcoxon rank sum test, and Spearman’s rank order correlation was performed to investigate whether the patient-specific performance of a biomarker was systematically linked to the patient’s median spike rate.

Furthermore, we conducted a permutation test to investigate whether spike–ripples performed significantly better than spikes regarding the distinction of SOZ from non-SOZ channels. First, for each channel, the spike–ripple and spike rates were transformed to biomarker-specific ranks, i.e., a spike–ripple and a spike rank, relative to the remaining channels, was assigned to each channel. It may be noteworthy that this step does not alter the two ROC curves because those are invariant to monotone increasing transformations of the measurement scale [37]. We then calculated the AUC difference based on our empirical data:

Diff(AUC)_empirical_ = AUC_spike-ripples_ − AUC_spikes_ (1)

Then, for each channel independently, we randomly swapped group labels, and again computed Diff(AUC) (‘Diff(AUC)_surrogate_’). This step was repeated 100,000 times to compute a distribution of Diff(AUC)_surrogate_. The performance of spike–ripples and spikes was considered significantly different if Diff(AUC)_empirical_ ranked above 97.5% or below 2.5% of all Diff(AUC)_surrogate_. A detailed investigation of the properties of such an approach can be found, e.g., in [38]. This procedure was also performed for the pAUC difference, and finally, in a slightly adapted fashion, for the functional vs. remaining non-SOZ classification.

## 3. Results

### 3.1. Patients and Channels

Nineteen subjects (9 females, 10 males; age: median 38 years, range 11–54 years, see Table 1 for more clinical data) met the inclusion criteria. The majority of our patients had a neocortical lesion, most often focal cortical dysplasia (*n* = 12 patients), whereas a mesiotemporal pathology was rare. Two patients had no clear epileptogenic lesion on their MRI. We analyzed recordings from all major lobes and a variety of functional regions, including primary motor (*n* = 15 patients) and sensory cortex (*n* = 13), Broca’s (*n* = 4) and Wernicke’s (*n* = 7) area. In patient 2, the grid did not cover the seizure onset zone (SOZ); this patient therefore had to be excluded from the patient-specific comparisons of SOZ and non-SOZ channels described below. Epilepsy surgery was performed in all but this patient, most often extended lesionectomy, and more than half of them (*n* = 11 patients) became completely seizure-free (median follow-up 6 months).

### 3.2. Biomarkers of the Seizure-Onset Zone

**Figure 1:**
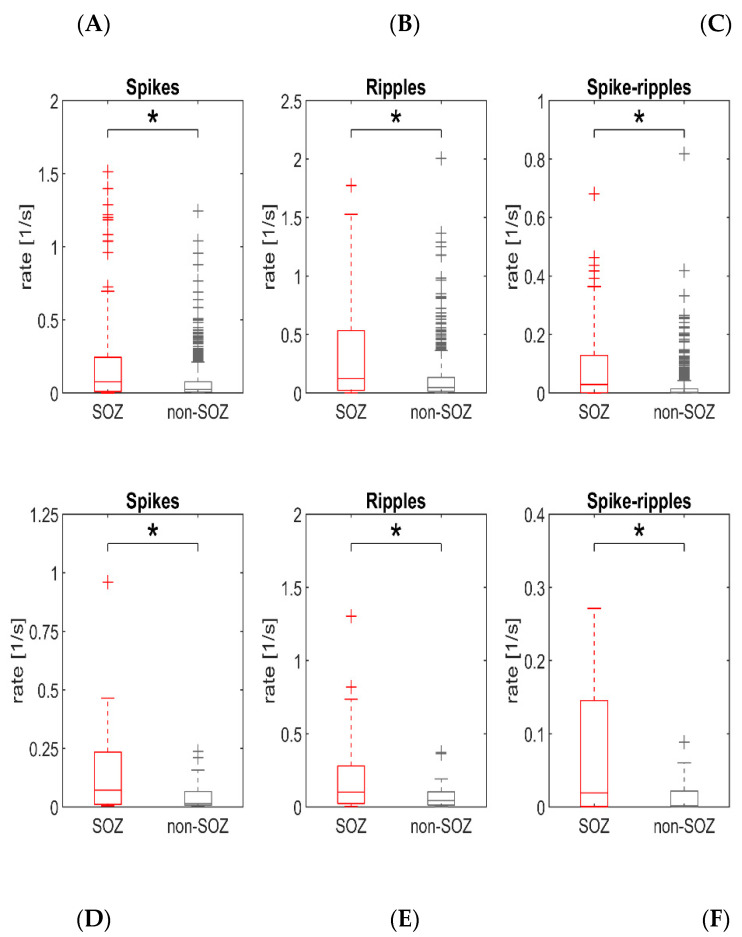
Interictal spikes, ripples and spike-ripples are biomarkers of the seizure onset zone (SOZ) in subdural grid recordings. (**A–C**) Channels pooled across patients. SOZ channels have higher rates of interictal spikes (**A**, *p* < 0.001), ripples (**B**, *p* < 0.001) and spike-ripples (**C**, *p* < 0.001) than non-SOZ channels. (**D**–**F**) Comparison at the level of individual patients. For each patient, and from both groups of channels, we considered the channel with the median rate as representative. Again, rates of interictal spikes (**D**, *p* = 0.001), ripples (**E**, *p* = 0.004) and spike-ripples (**F**, *p* < 0.001) were higher inside the SOZ.

Interictal spikes, ripples and spike–ripples have each been suggested as biomarkers of epileptogenic tissue. First, we therefore investigated whether this applies also to our particular dataset, which only contained recordings from the neocortex and with subdural grid electrodes. Comparing all identified channels, pooled across patients, it was revealed that the seizure onset zone generated significantly higher rates of interictal spikes (*p* < 0.001, Wilcoxon rank sum test; SOZ: *n* = 258 channels, non-SOZ: *n* = 635 channels), ripples (*p* < 0.001) and spike–ripples (*p* < 0.001) than the remaining non-SOZ regions (Figure 1). To perform a similar analysis at the level of patients, we also compared each subject’s median SOZ channel, i.e., the channel with a median rate from this subject’s SOZ channels, to the median non-SOZ channel. Again, the rates of interictal spikes (*p* = 0.001, Wilcoxon signed-rank test; *n* = 18 patients), ripples (*p* = 0.004) and spike–ripples (*p* < 0.001) were higher inside the SOZ. In summary, these findings suggest that the analysis of all three biomarkers in subdural grid electrode recordings reveals localizing information on seizure-generating neocortical regions.

### 3.3. Systematic Comparison of Diagnostic Value

**Figure 2:**
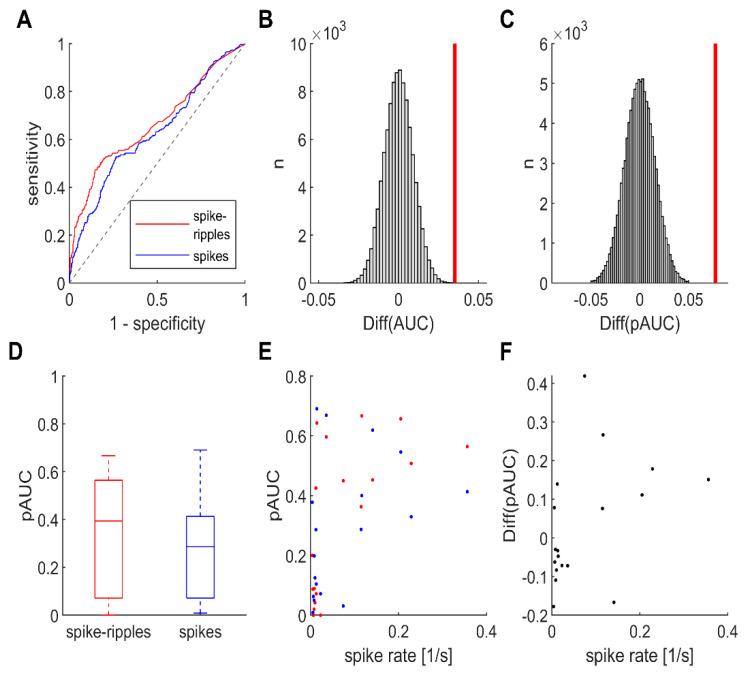
Interictal spike-ripples identify SOZ channels slightly but significantly better than spikes. (**A**) ROC curves, SOZ vs. non-SOZ classification, channels pooled across patients. The AUC for spike-ripples (red line, 0.664) was slightly higher than for all spikes (blue line, 0.628). (**B**) To investigate whether this difference was significant, we applied permutation-based hypothesis testing. The probability of obtaining an AUC difference that is as high as or higher than our empirically measured value (bold vertical line) by chance (grey bars represent surrogate data) was less than 5 % (two-tailed *p* < 0.001). (**C**) Same analysis for pAUC (between 85 and 100 % specificity), as applied in [32]. Again, spike ripples classified significantly better (*p* < 0.001). (**D**) At the level of individual patients, there was no significant difference between the pAUC for spike-ripples and spikes (*p* = 0.29). Note the broad range of values, reflecting that the SOZ could be nicely delineated in several patients, whereas performance was poor in others. (**E**) Both biomarkers classify better in patients with many interictal spikes. There was a significant correlation between our patients’ median spike rate and their pAUC, both for spike-ripples (red, *p* = 0.002) and spikes (blue, *p* = 0.02). Each dot corresponds to one patient. (**F**) Spike-ripples are a better classifier in patients with many spikes. There was a significant correlation between our patients’ median spike rate and their pAUC difference between spike-ripples and spikes (*p* = 0.042).

To compare how reliably these biomarkers could generally distinguish SOZ from non-SOZ channels, we analyzed their receiver operating characteristic (ROC) curves (Figure 2A). The area under the curve (AUC), as the standard parameter quantifying performance, was the highest for spike–ripples (0.664), intermediate for ripples (0.640) and lowest for spikes (0.628). Noticing that the shape of these curves was especially different for high specificities, we also computed the partial AUC (pAUC) between 100% and 85%, which was also highest for spike–ripples (0.252), intermediate for ripples (0.244) and lowest for spikes (0.181). Permutation-based testing revealed that the difference between spike–ripples and spikes was significant, both for AUC (*p* < 0.001; Figure 2B) and pAUC (*p* < 0.001; Figure 2C). It can thus be concluded that in our dataset, focusing on interictal spikes with a co-occurring ripple oscillation improved our identification of SOZ channels significantly.

### 3.4. Diagnostic Value at the Level of Individual Patients

Considering that the impact of a biomarker on clinical decision-making depends on its performance in individual patients, we also computed patient-specific ROC curves, and the corresponding AUC and pAUC (Figure 2D). For both spike–ripples and spikes, a broad range of values was obtained, reflecting that the SOZ could be nicely delineated in several patients, whereas performance was poor in others. In summary, there was no significant difference between spike–ripples and spikes in our group of patients (AUC: *p* = 0.71, pAUC: *p* = 0.29; Wilcoxon signed-rank test; *n* = 18 patients). There was a significant correlation between our patients’ median spike rate and their pAUC (Figure 2E), both for spike–ripples (rho = 0.68, *p* = 0.002; Spearman’s rank order correlation) and spikes (rho = 0.54, *p* = 0.02). Moreover, the patient-specific pAUC difference between spike–ripple- and spike-based classification correlated with the subject’s median spike rate (rho = 0.48, *p* = 0.042). These results suggest that both biomarkers perform better in patients with many spikes and that in these individuals, spike–ripples may be superior.

### 3.5. ‘Pure’ Ripples in Eloquent Cortex

**Figure 3:**
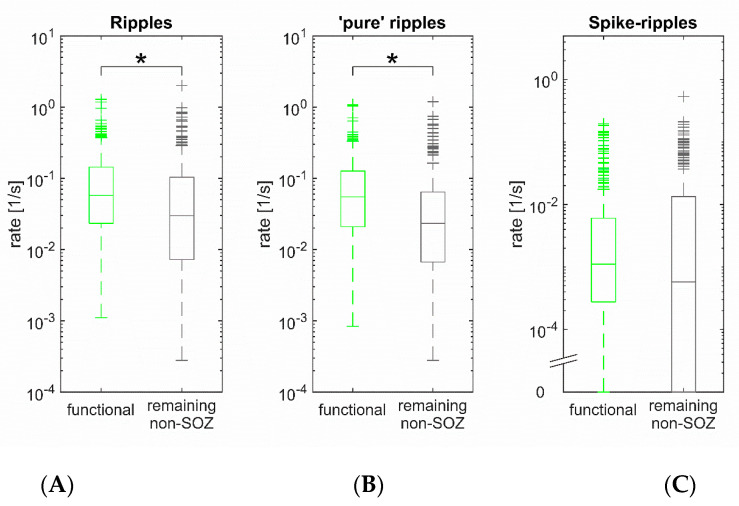
Eloquent cortex generates ‘pure’ ripples. (**A**) Ripple rate was higher (*p* < 0.001) in eloquent cortex than in remaining non-SOZ channels. (**B**) This difference was due to the fact that eloquent cortex generates significantly more ‘pure’ ripples (*p* < 0.001). (**C**) Spike-ripple rates, in contrast, were not significantly different (*p* = 0.87).

Our main aim was to investigate whether ‘pure’ ripples, i.e., those occurring independently from spikes, are more frequent in the eloquent neocortex. First, we therefore identified channels with a functional response to stimulation and compared them to all remaining non-SOZ channels. The ripple rate in general was higher in the eloquent cortex than in the remaining non-SOZ channels (*p* < 0.001; Wilcoxon rank sum test; eloquent cortex: *n* = 311 channels, non-SOZ remaining: *n* = 282 channels; Figure 3), and this difference was due to a significant difference in ‘pure’ ripples (*p* < 0.001; median rates 3.3/min vs. 1.4/min). Spike–ripple rates, in contrast, were not significantly different (*p* = 0.87). To perform a similar analysis at the level of patients, we then compared each subject’s median channels between the two groups, in analogy to the SOZ vs. non-SOZ comparison described above. Again, ripples (*p* = 0.03, Wilcoxon signed-rank test; *n* = 19 patients) and specifically ‘pure’ ripples (*p* = 0.02) were more frequent in eloquent cortex, whereas spike–ripples did not differ significantly (*p* = 0.98). It can thus be concluded that neocortical ‘pure’ ripples likely reflect physiologic fast oscillatory activity.

### 3.6. Delineation of Eloquent Cortex—Across and in Individual Patients

**Figure 4:**
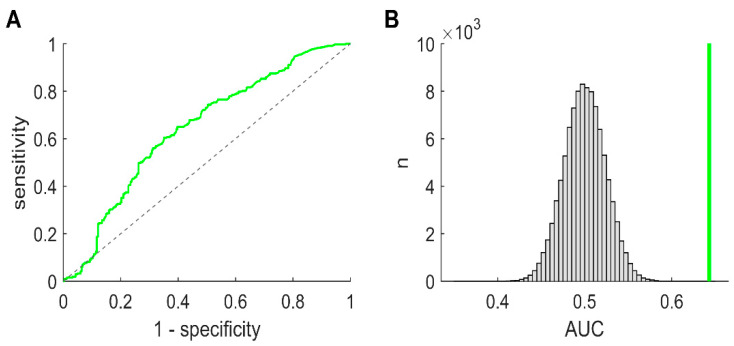
Delineation of eloquent neocortex based on ‘pure’ ripples—across patients. (**A**) ROC curve, functional vs. remaining non-SOZ classification, channels pooled across patients. (**B**) To investigate whether ‘pure’ ripples identify eloquent regions significantly better than chance, we again applied permutation-based testing. The probability of obtaining an AUC that is as high as or higher than our empirically measured value (bold vertical line) by chance (grey bars represent surrogate data) was less than 5% (two-tailed *p* < 0.001).

**Figure 5:**
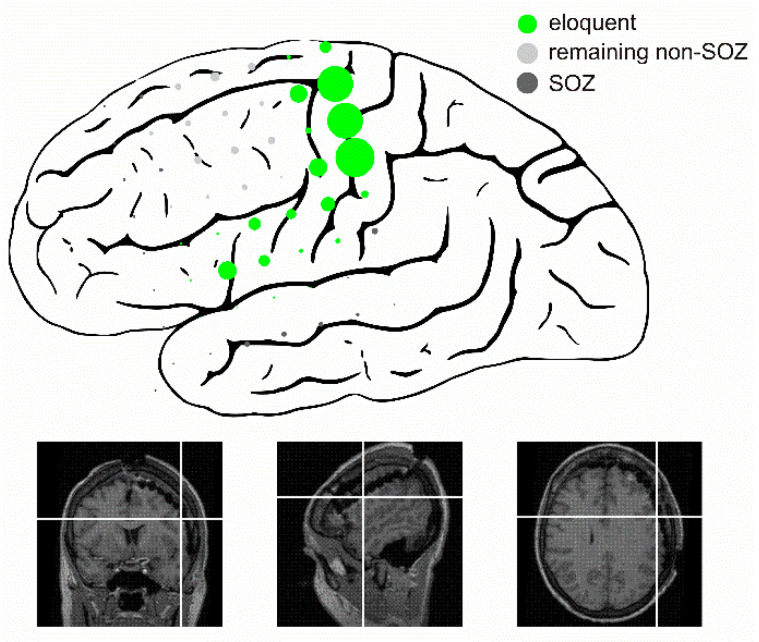
Delineation of eloquent neocortex based on ‘pure’ ripples in patient 9. Schematic illustrates approximate location of grid electrodes, MRI sections below are displayed for reference. Each filled circle represents one bipolar channel, with its radius proportional to its ‘pure’ ripple rate. Note that ‘pure’ ripples were particularly frequent in clearly functional neocortex, identified based on electrical stimulation. In this patient, these regions correspond nicely to the typical location of Broca’s area, primary motor and sensory cortex.

Finally, we explored whether ‘pure’ ripples might be of value regarding the delineation of the functional neocortex in pre-surgical patients with epilepsy. To this end, we also computed a receiver operating characteristic (ROC) curve for the functional vs. remaining non-SOZ classification (Figure 4A). Across patients, ‘pure’ ripples identified functional tissue significantly better than chance (AUC = 0.64; *p* < 0.001, permutation test; Figure 4B). In line with these findings, we found that ‘pure’ ripples nicely delineated an eloquent neocortex in some patients (Figure 5). Our data thus suggest that ‘pure’ ripples could be of value, in addition to electrical stimulation, if functional regions have to be mapped—but to what extent they are a reliable tool clearly has to be examined in a larger systematic study. 

## 4. Discussion

The main novel finding of this study is that in interictal recordings from subdural grid electrodes, ’pure’ ripples are more frequent in the unequivocally functional cortex, identified based on electrical stimulation. Moreover, we report that spike–ripples identify seizure onset better than spikes, which underlines that they are highly pathologic. Various aspects of these findings shall be discussed in detail below.

### 4.1. ‘Pure’ Ripples: A Reproducible Marker of Eloquent Neocortex

Our main finding is that ‘pure’ ripples, i.e., ripples not associated with spikes, occur significantly more often in the eloquent neocortex than in remaining non-SOZ areas. The rates we report are very similar to a recent study on physiologic HFOs, which also found the highest rates in typically eloquent regions [39]. This may be noteworthy because the dataset we analyzed was different in several important aspects, and because we applied a different algorithm for automated detection. Furthermore, we provide solid evidence for the concept that our ‘pure’ ripples are truly physiological because (1) they were recorded from unequivocally functional neocortex, as demonstrated by stimulation, and because (2) the difference between eloquent and remaining non-SOZ channels was only significant for ‘pure’ ripples, and not for spike–ripples. Together with a previous study on specific coupling to slow waves [23], these data suggest that HFOs in ‘healthy’ tissue are generated based on distinct mechanisms. Our recordings covered a variety of regions, including primary motor and sensory cortex, Broca’s and Wernicke’s area. It thus seems unlikely that ‘pure’ ripples are directly linked to only one specific function, but future studies will have to investigate such questions in detail. Another aspect is that the stimulation as such suppresses HFOs [40], and it may be interesting to explore if this effect applies particularly to physiological HFOs. Anyway, it can be concluded that ‘pure’ ripples are indeed a marker of eloquent regions, and that they probably reflect physiologic brain activity.

### 4.2. Delineation of Epileptogenic Tissue AAre Spike–Ripples a Better Biomarker?

We report that interictal spikes with a co-occurring ripple oscillation identify the seizure onset zone (SOZ) channels significantly better than spikes. This finding is in line with previous work [28,29,30] and the stereotyped coupling of HFOs to spike-slow waves may also be characteristic of epileptogenic areas [8]. At the level of individual patients, however, there was no significant difference between the two biomarkers, which is consistent with a previous study analyzing stereotactic electroencephalography in 30 patients [32]. Therefore, in summary, do spike–ripples provide additional value, or not? It should be acknowledged that both ours and the previous study had limited power to detect differences at the patient level-and our channel-based analysis does suggest that spike–ripples are slightly—but significantly—superior. Furthermore, our study underlines that patient-specific features must be taken into account: while performance differed substantially between individuals, spike–ripples were particularly better if high specificity was required, and in patients with many spikes. In conclusion, spike–ripples may not be suited to delineate non-epileptogenic areas but are superior if clearly pathologic tissue has to be identified.

### 4.3. Interactions between Spikes and Ripples

High-frequency oscillations (HFOs) sometimes co-occur with spikes, and interactions between these two kinds of activity have been examined since the first reports on HFOs in humans [41,42,43]. A potential confounder in any such study is that high-pass filtering of sharp transients may artificially generate ‘false’ ripples [44]. Such effects can be minimized by using filters with a finite impulse response [45], implementing a criterion of, e.g., at least four oscillatory cycles [5,45], or by performing a time–frequency decomposition as in the detection algorithm we applied [16,35]. Despite these methodological challenges, several lines of evidence suggest that spike–ripples are a distinct pathophysiological phenomenon: ripples riding on spikes are frequently visible even in unfiltered traces, as recent studies have revealed their value in different clinical scenarios [28,31], and we have reported that spikes with HFOs have a distinct single-neuron correlate [46]. The current study demonstrates that spike–ripples and ‘pure’ ripples are two distinct kinds of activity, the former being epileptic and the latter being physiological, and which clearly underlines the relevance of understanding interactions between spikes and HFOs.

### 4.4. Limitations and Outlook

This study is limited in several ways. Focusing on subdural grid recordings from neocortical tissue, we obtained a relatively homogeneous dataset, but this also implies that our conclusions do not necessarily apply to other regions, such as the mesial temporal lobe. Another issue is that spikes and ripples were detected by an algorithm that had not been developed at our center, based on data from depth electrodes recorded with a setup from a different company. We decided not to modify the default settings of this detector-and one might speculate that if we had done so, its accuracy and thus the performance of our biomarkers, would have been improved. On the other hand, we thus demonstrated that HFO analysis is feasible without time-consuming visual identification, ‘optimization’ or even the new development of an automated algorithm. In addition, the distinction of epileptic and physiologic HFOs may be improved by integrating our coupling-based strategy in a multivariate classifier, which also takes other HFO features or interactions with sleep into account. Such approaches promise to open up new avenues for a variety of research questions, in both epilepsy and cognitive science.

## 5. Conclusions

This study suggests that ‘pure’ ripples are physiological and a marker of eloquent neocortex. Spike–ripples, in contrast, provide additional value regarding the delineation of epileptogenic tissue. Considering that both types of events were detected with open source software that was not developed at our center, our approach should permit to efficiently tackle a variety of research questions, in both epilepsy and cognitive science.

## Figures and Tables

**Table 1:** Clinical data. Abbreviations: AHE, amygdalohippocampectomy; ATL, anterior temporal lobectomy; EL, extended lesionectomy; F, frontal lobe; f, female; FCD, focal cortical dysplasia; L, left; m, male (second column)/months (last column); MST, multiple subpial trans-sections; P, parietal lobe; O, occipital lobe; R, right; s/p, status post; T, temporal lobe; y, years.

ID	Sex	Age (y)	MRI	Grid Coverage	Eloquent Cortex	AEDs	Surgery	Outcome, Engel Class (Follow-Up)
1	m	21	S/p resection pilocytic astrocytoma, gliosis temporobasal L	T	Wernicke	OXC	EL	Ia (1 y)
2	f	51	Hippocampal sclerosis L	T, F	Wernicke, motor	LEV, PGB	AHE	Ia (1 y)
3	f	28	no clear epileptogenic lesion	F, T, P, O	Motor, sensory, Wernicke	OXC, PGB	none	N/A
4	m	11	FCD frontoparietal R	F, P	Motor, sensory	LEV, OXC, ZNS	EL	Ia (3 m)
5	m	41	S/p resection ganglioglioma parietal L	P, T, F	Wernicke, motor, sensory	ZNS, PHT	EL	Ib (3 m)
6	f	21	FCD frontal R	F, T, P	Motor, sensory	OXC, TPM, CLB	EL	Ia (3 m)
7	m	41	FCD frontocentral R	F, P	Motor, sensory	LTG, CLB	EL + MST	Ia (3 m)
8	m	39	Hippocampal sclerosis L, FCD temporal L	T, F	Motor	LEV	EL + AHE	Ia (3 m)
9	f	48	FCD frontal L	F, P	Motor, sensory, Broca	OXC, PHT, DZP	EL	IIb (1 y)
10	m	54	Gliosis frontal L	F	Motor, Broca	OXC, LEV	EL	Ia (6 m)
11	f	33	FCD frontal R	F, P	Motor, sensory	OXC, CLB	EL	Ia (1 y)
12	f	27	S/p AHE, FCD temporal L	T	Wernicke	OXC, LCM	ATL + MST superior temporal gyrus	IVb (1 y)
13	m	17	FCD temporal R	T, P	Sensory	LEV, OXC	EL + AHE	Ia (2 y)
14	f	14	FCD occipitotemporal L	T	Wernicke	OXC, LTG, PHT	MST	IVb (1 y)
15	f	15	FCD frontal R	F, P	Motor, sensory	STM, LTG, CLB	EL	Ia (3 m)
16	f	38	FCD frontal L	F, P	Motor, sensory, Broca	LTG, LCM, TPM, CLB	partial resection frontal lobe	IIIa (6 m)
17	m	33	no clear epileptogenic lesion	F, P	Motor, sensory	LTG, RUF, CLB	MST	IIIa (6 m)
18	m	22	FCD frontal L	F, T, P, O	Motor, sensory, Broca, Wernicke	OXC, LTG, CLB	EL	IIIa (1 y)
19	m	14	Gliosis frontal R	F, P	Motor, sensory	LEV	EL	Ia (3 m)

## Data Availability

Not Applicable.
